# Supplementary material for: Genetic correlations reveal the shared genetic architecture of transcription in human peripheral blood
Source: Nat Commun. 2017 Sep 7;8:483. doi: 10.1038/s41467-017-00473-z (PMC5589780; doi:10.1038/s41467-017-00473-z)
Supplement: Supplementary file 1 — Supplementary Information [file 41467_2017_473_MOESM1_ESM.pdf]

Description of Supplementary Files

File Name: Supplementary Information
Description: Supplementary Figures and Supplementary Tables

File Name: Supplementary Data 1
Description: Significant genetically correlated probes (Bonferroni) Table of 556 significant genetically correlated probe pairs (Bonferroni, $p < 1.81*10^{-8}$). Of these, 506 (91%) map to different genes, and 427 (77%) pairs are located on different chromosomes and 128 (23%) are on the same chromosome.

File Name: Supplementary Data 2
Description: Significant genetically correlated probes (FDR) Table of 14,991 significant genetically correlated probe pairs (FDR < 0.05). Of these, 14,020 (93.5%) pairs are located on different chromosomes, 7,886 have a positive r_G and 7,105 have a negative r_G,

File Name: Supplementary Data 3
Description: Genetically correlated probes with shared eSNPs in LIFE-Heart data. Table of 934 significant (Bonferroni) genetically correlated probe pairs with a shared eSNP from the results of the LIFE-Heart study. The data shows the identifier of the most significant shared eSNP, and the eSNP pand beta values for both probes in a probe-pair.

File Name: Supplementary Data 4
Description: Shared eSNPs replicated in CAGE data. Table of eSNPs identified as significant in LIFE-Heart data and were also present in the CAGE eQTL data. For the 651 out of 934 LIFE-Heart eSNPs found in the CAGE summary data, 100% of the LIFE-Heart eSNPs and genetically correlated probe pairs replicate with 100% matched allelic direction.

File Name: Peer Review File

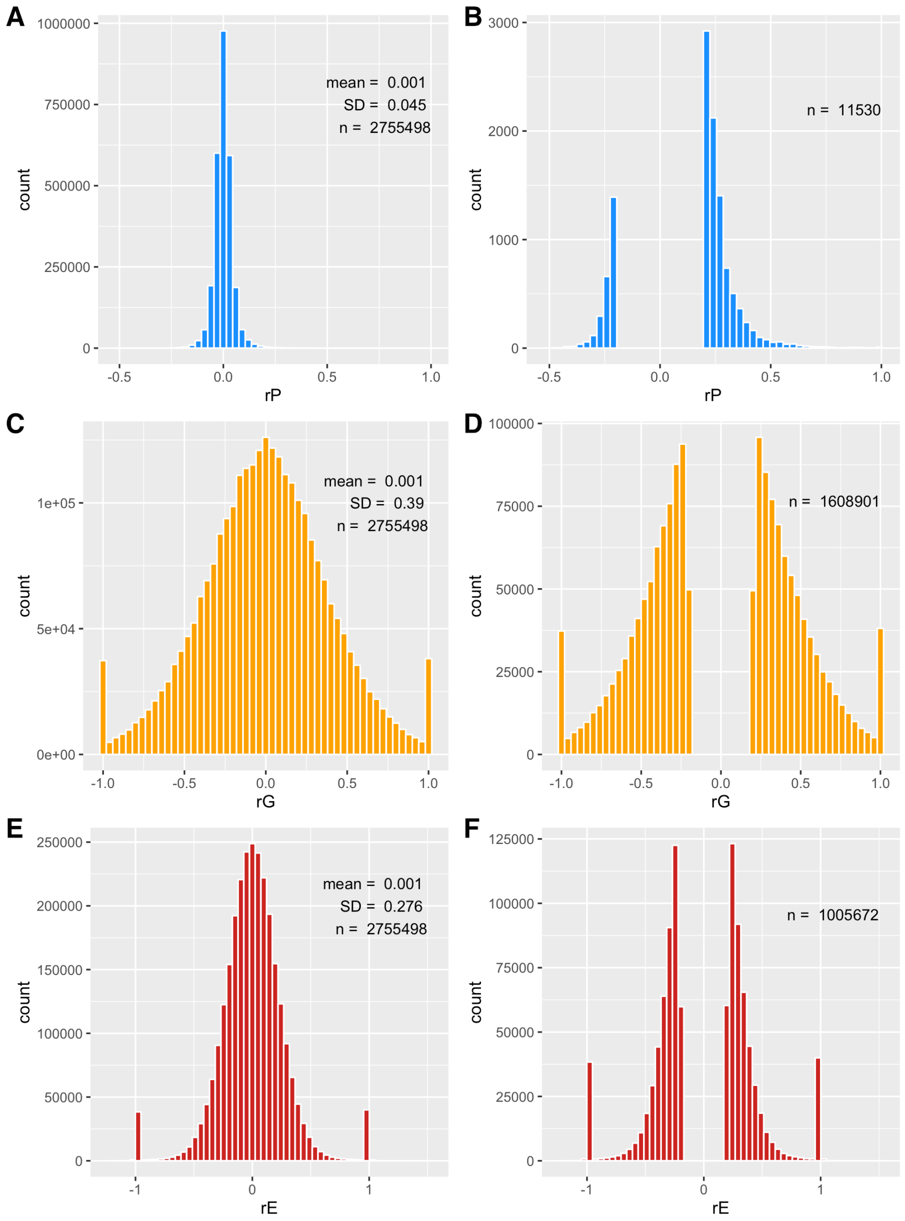

Supplementary Figure 1: Distribution of r_P, $\hat{r}_G$ and $\hat{r}_E$ for transcript pairs exceeding the transcript heritability threshold. Phenotypic correlations (r_P) were calculated for all 2,755,498 transcript pairs with heritability $\geq$ 0.25 (A). Closer inspection of the distribution for pairs $|r_P| \geq$ 0.2, shows a bias towards positively correlated transcript pairs (B). Genetic correlations ($\hat{r}_G$) and environmental correlations ($\hat{r}_E$) estimated for all transcript pairs (C and E), and those $|\hat{r}_G|$ or $|\hat{r}_E| \geq$ 0.2 (D and F), are symmetrical about the mean.

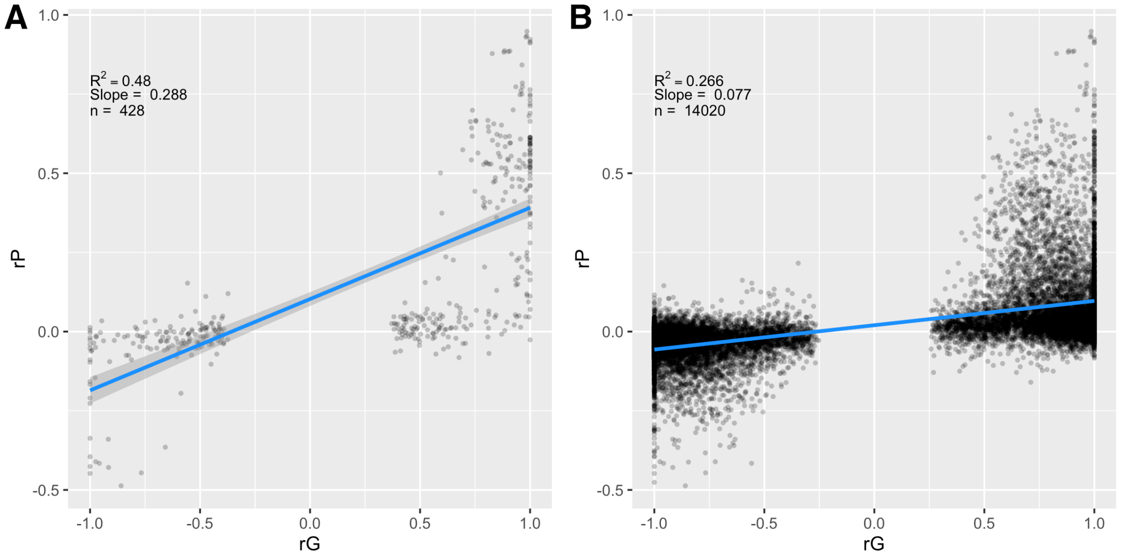

Supplementary Figure 2: The relationship between $\hat{r}_G$ and r_P for (A) 428 (Bonferroni $p < 1.81 * 10^{-8}$) and (B) 14,020 (FDR 0.05) significant *trans* transcript pairs, reveals a bias towards same-signed directionality.

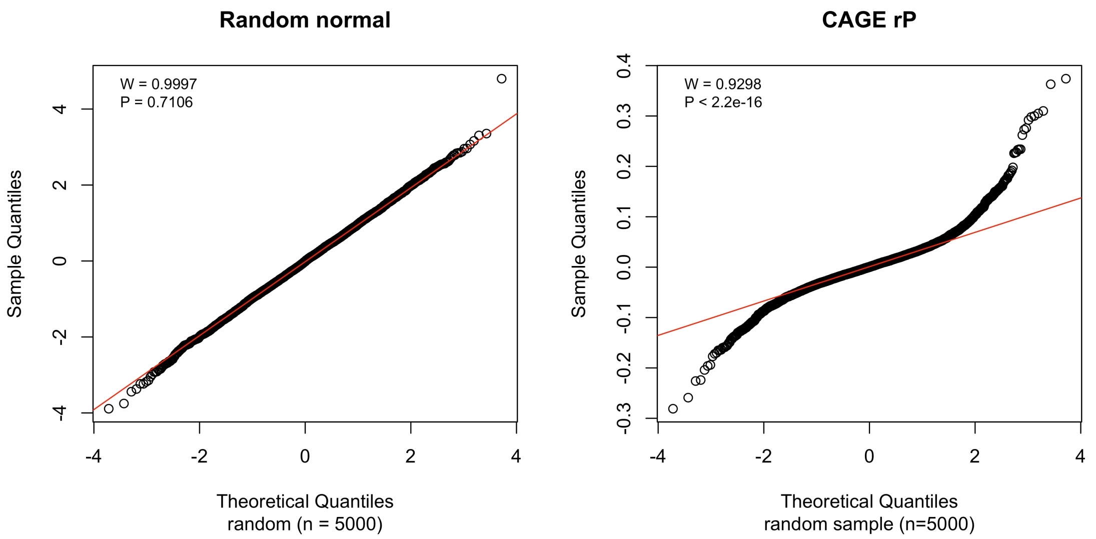

Supplementary Figure 3: Quantile-quantile plots of the r_P distribution compared to a random normal distribution. The r_P value for 5,000 transcript pairs was randomly sampled from the complete dataset tested for normality using the Shapiro-Wilk test. A dataset of 5,000 random values was generated as a normal distribution control.

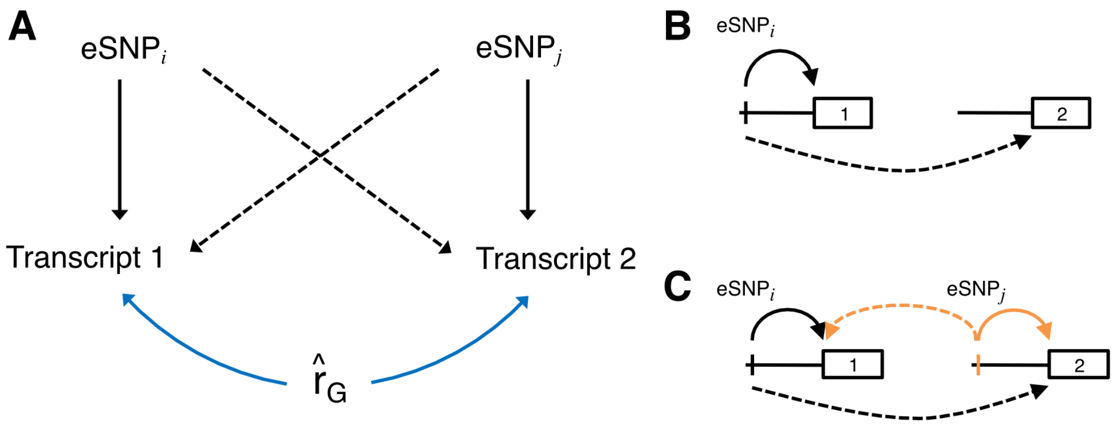

Supplementary Figure 4: Schematic outlining the discovery of shared eSNP effects on correlated transcript pairs. Panel A: The effect of the top eSNP for each transcript, eSNP$_i$-transcript$_1$ or eSNP$_j$-transcript$_2$ (shown by black arrows), was tested for a shared effect on the other transcript, such that eSNP$_i$-transcript$_2$ or eSNP$_j$-transcript$_1$ represents a shared eSNP between transcript 1 and 2, or transcript 2 and 1 (dashed black arrows). The $\hat{r}_G$ between transcripts pairs is represented by a blue curved arrow. Panel B: A unidirectional model where the top eSNP for transcript 1 regulates its expression in *cis* and alters the expression of transcript 2 in *trans*. Panel C: A bi-directional model in which the top eSNP for transcript 1 alters the expression of both transcript 1 and transcript 2, and the top eSNP for transcript 2 (different from the first eSNP, shown in orange) also alters the expression of both transcript 1 and transcript 2.

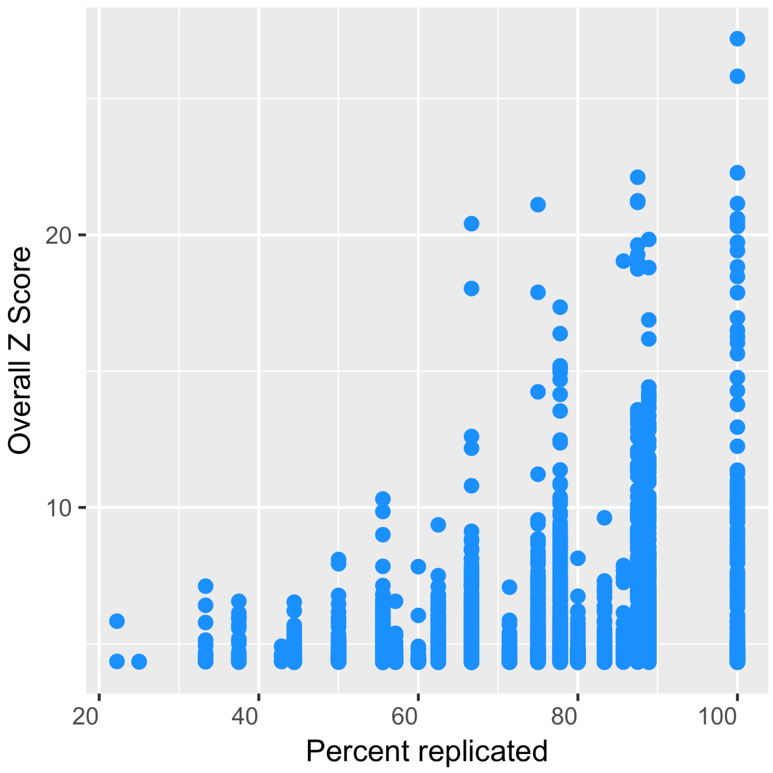

Supplementary Figure 5: Replication of large-effect trans-eQTLs in multiple datasets. Percentage of trans-eQTLs with large effects, shown as absolute Z-score, that were replicated in multiple blood eQTL datasets. Trans-eQTL data (FDR 0.5) was obtained from the Westra *et al.* 2012 paper and its associated web browser (http://genenetwork.nl/bloodeqtlbrowser/).

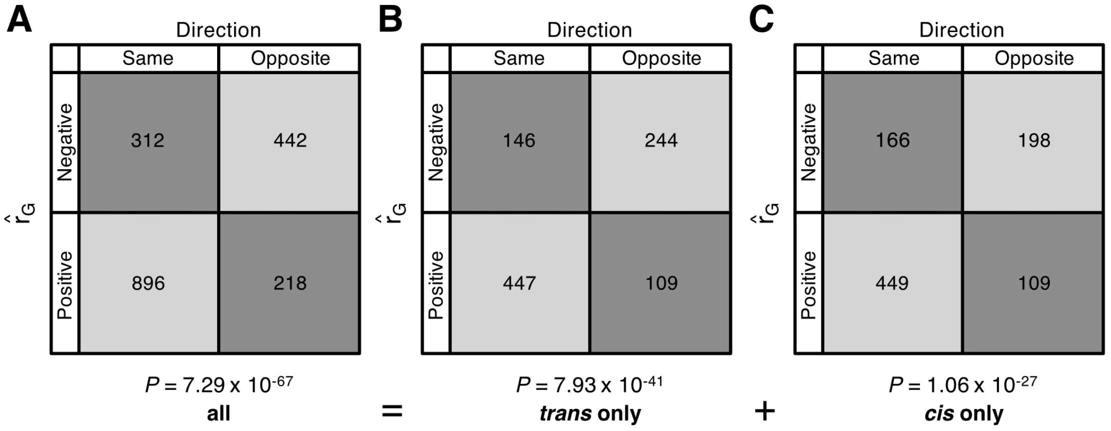

Supplementary Figure 6: A chi-square punnet detailing the number of shared $\hat{r}_G$ direction and shared eSNP effect β for the 934 eSNP pairs significant at a Bonferroni threshold. These data reveal a bias towards transcripts with a positive $\hat{r}_G$ and same allelic direction of effect (bottom-left vs. bottom-right), and transcripts with a negative $\hat{r}_G$ and opposite allelic direction of effect (top-right vs. top-left). Panel A contains results for all pairs, and panels B and C contain *trans*- and *cis*-only pairs, respectively. The light grey shading represents the expected direction of the $\hat{r}_G$/eSNP β, and dark grey represents $\hat{r}_G$/eSNP β relationships not in the expected direction.

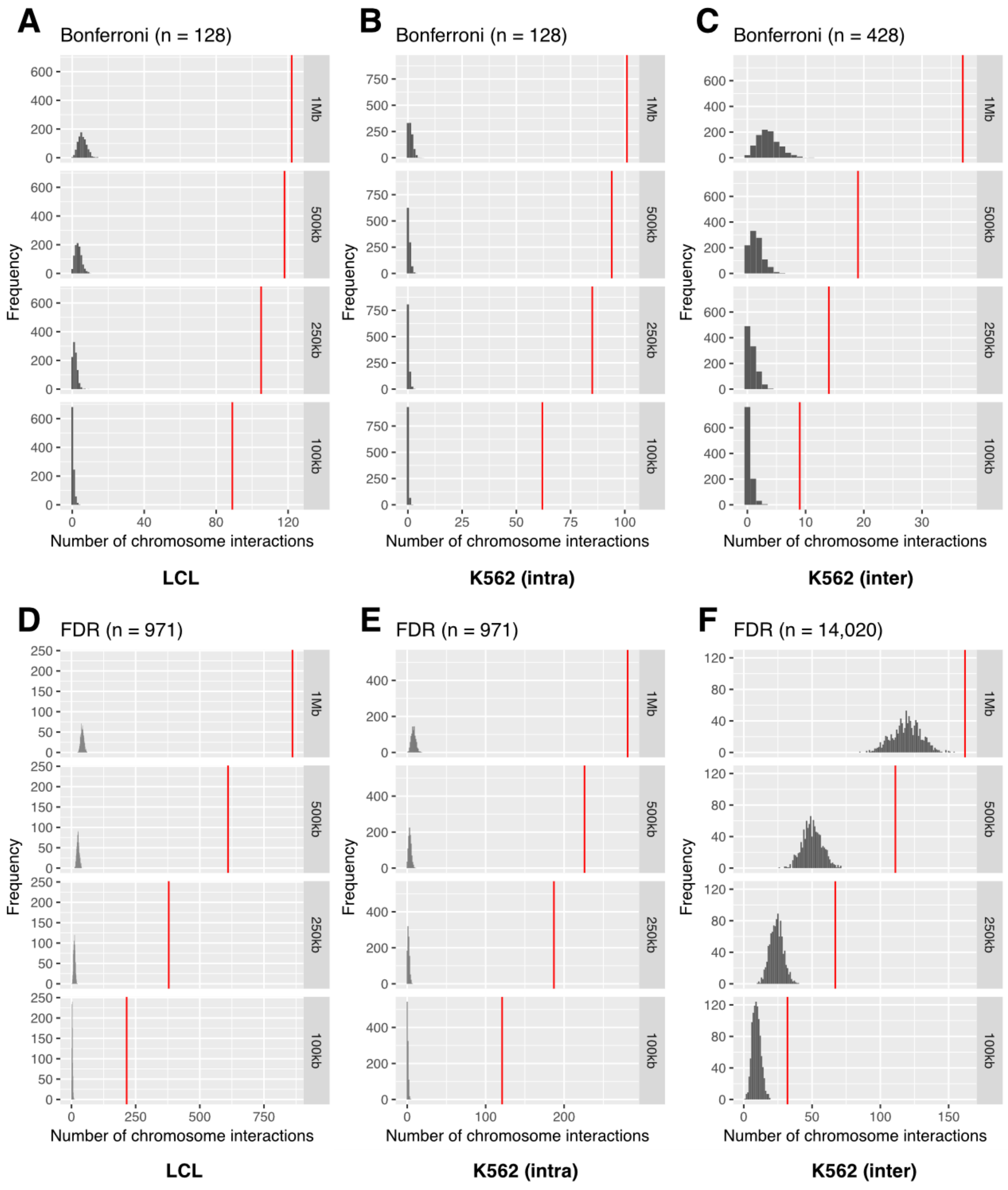

Supplementary Figure 7: Number of overlaps between genetically correlated transcript pairs and known regions of interacting chromatin in LCL and K562 cells. The number of transcript pairs, from (A-C) the Bonferroni-corrected ($p < 1.81 * 10^{-8}$) $\hat{r}_G$ subsets (n = 128 intrachromosomal; n = 428 interchromosomal), and the study-wide FDR of 0.05 subset (D-F; n = 971 intrachromosomal; n = 14,020 interchromosomal), that overlap known LCL and K562 chromatin interaction sites in windows of 100kb, 250kb, 500kb or 1Mb are shown by red lines. The null distribution represented as a grey histogram was obtained by randomly sampling each dataset 1,000 times for each window size.

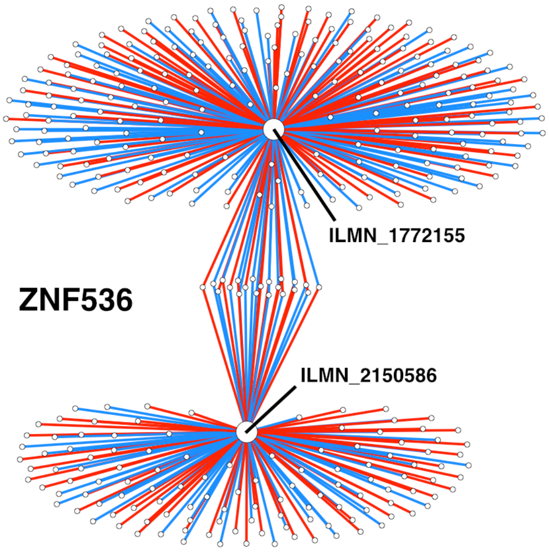

Supplementary Figure 8: Network graph of genetically correlated transcript pairs. The connections between 314 transcripts that are directly genetically correlated with *ZNF536* and are more than 4σ ($|\hat{r}_G| \geq$ 0.96) from the mean (H_0: $\hat{r}_G$ = 0), are shown with blue and red lines representing positive (blue) and negative (red) r_G estimates.

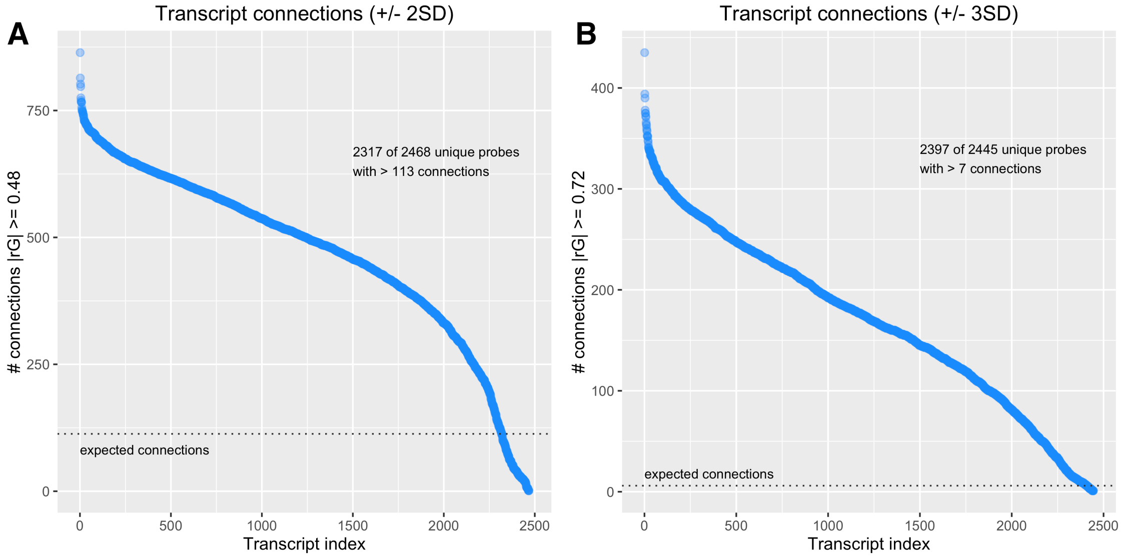

Supplementary Figure 9: Number of observed vs expected connections for transcripts with $\hat{r}_G$ estimates above 2 (A) and 3 (B) standard deviations from the mean of the null distribution (H_0: $\hat{r}_G$ = 0). Expected number of connections (dotted line) was calculated as: (1-SD%) x # unique transcripts, where SD% = 0.954 (2σ) or 0.997 (3σ).

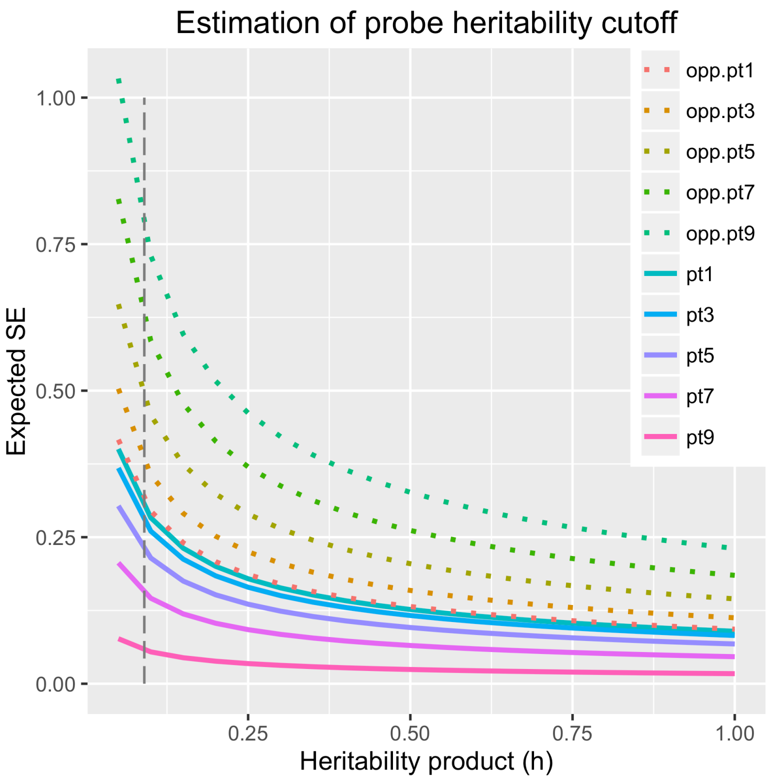

Supplementary Figure 10: Estimation of probe heritability cutoff. We termed the multiplied heritability values of two transcripts, $H = h^2_{Gi}h^2_{Gj}$, where $h^2_{Gi} == h^2_{Gj}$. This was plotted against the expected $\hat{r}_G$ standard error, and r_P and $\hat{r}_G$ values of same- or opposite-sign were tested to identify combinations that would result in a large SE. A threshold of H = 0.0625 (grey dashed line) was selected to minimise the SE for all positive r_P and $\hat{r}_G$ combinations, as well as opposite-signed combinations with small values. This threshold excluded opposite-signed combinations ¿ 0.5 which generated large SE values. Expected SE was calculated as shown below, where x is 1/square root of the GRM off-diagonals and N is the population size:

$$SE(\hat{r}_G) \approx \frac{x}{N}\sqrt{\frac{(1 - r_G r_P)^2 + (r_G - r_P)^2}{H}}$$

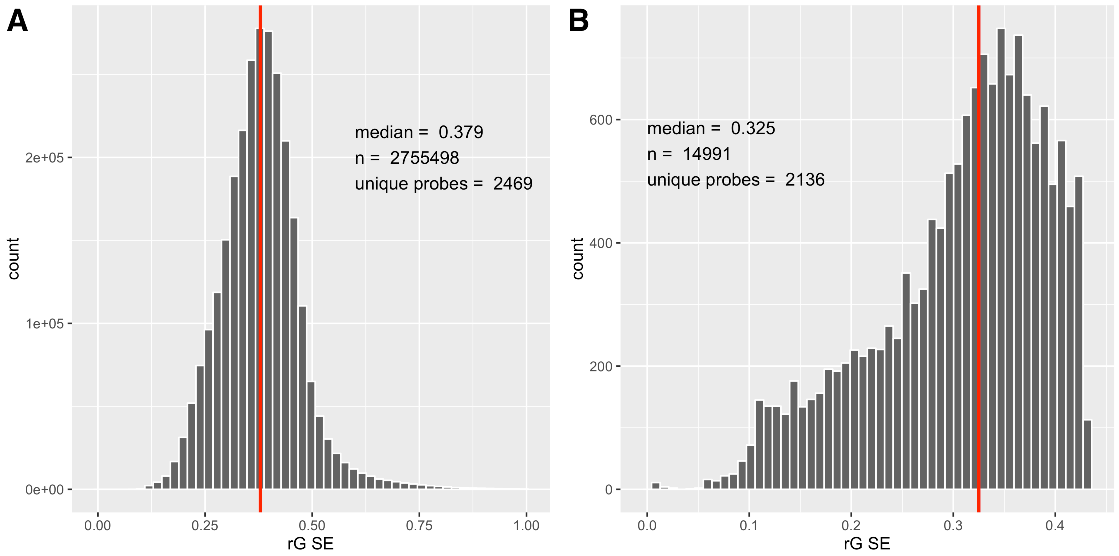

Supplementary Figure 11: Distribution of $\hat{r}_G$ standard error for 2,755,498 (A) and 14,991 (B) genetically correlated transcript pairs exceeding the transcript heritability threshold of 0.25. The red vertical line represents the median standard error.

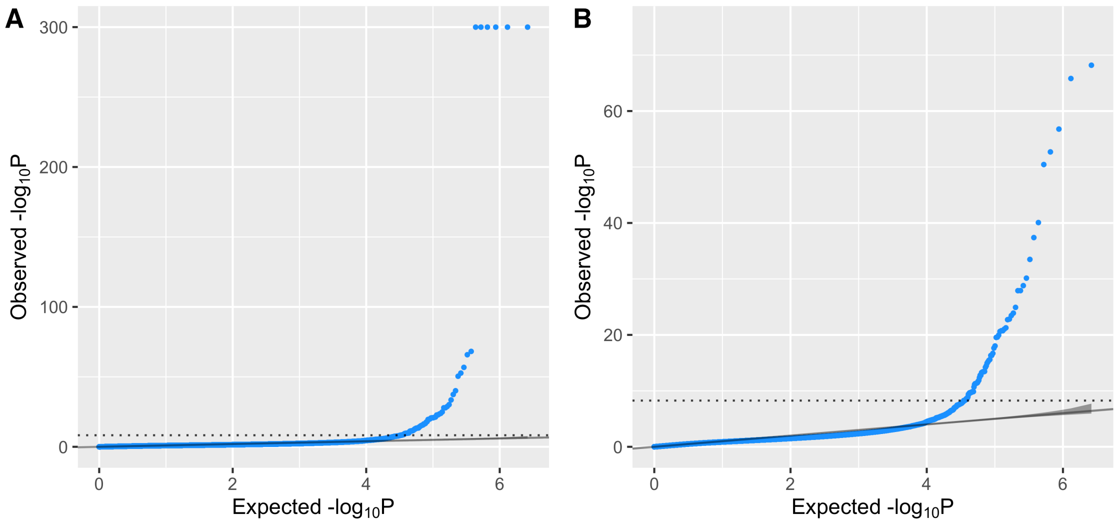

Supplementary Figure 12: A quantile-quantile plot shows the observed (y-axis, -log10 scale) and expected p-values (x-axis, -log10 scale) for each *trans*-only transcript pair in the analysis as determined by a chi-square test. Panel A: The complete *trans*-only subset where transcript pairs are not located on the same chromosome. Panel B: Zoomed plot of *trans*-only pairs to show $p \geq 1 * 10^{-100}$. The dotted line marks the multiple testing threshold (Bonferroni: $1.81 * 10^{-8}$).

Cell type	Subset	Location	Region	Obs	Exp	Fold change	P value
LCL	Bonf.	intra	1Mb	122	5.60	21.70	<0.001
			500kb	118	3.40	35.00	<0.001
			250kb	105	1.50	69.50	<0.001
			100kb	89	0.40	216.00	<0.001
LCL	FDR	intra	1Mb	861	42.10	20.50	<0.001
			500kb	610	25.60	23.90	<0.001
			250kb	379	11.30	33.50	<0.001
			100kb	215	3.00	70.60	<0.001
K562	Bonf.	intra	1Mb	122	1.20	105.10	<0.001
			500kb	118	0.50	253.20	<0.001
			250kb	105	0.20	470.90	<0.001
			100kb	89	0.10	1236.10	<0.001
K562	FDR	intra	1Mb	861	8.10	105.70	<0.001
			500kb	610	3.40	177.10	<0.001
			250kb	379	1.70	228.60	<0.001
			100kb	215	0.60	349.00	<0.001
K562	Bonf.	inter	1Mb	37	3.60	10.20	<0.001
			500kb	19	1.50	12.80	<0.001
			250kb	14	0.70	19.20	<0.001
			100kb	9	0.30	32.10	<0.001
K562	FDR	inter	1Mb	162	119.20	1.40	<0.001
			500kb	111	50.10	2.20	<0.001
			250kb	67	24.00	2.80	<0.001
			100kb	32	9.30	3.50	<0.001

Supplementary Table 1: Number of observed transcript pairs overlapping with known LCL and K562 chromatin interactions. Six subsets from two data sets were analysed containing intra- or interchromosomal transcript pairs for four window sizes (100kb, 250kb, 500kb or 1Mb). The analysis was also performed for 128 and 971 intrachromosomal pairs (Bonferroni and FDR, respectively) and 428 or 14,020 interchromosomal transcript pairs. Expected values are the mean of 1,000 random permutations. p-values were calculated using the best empirical p-values of the observed value against the ranked expected values.

	% intra	% inter	% total neg	% intra	% inter	% total pos
all	5.06	94.94	100	5.08	94.92	100
2σ	5.13	94.87	100	5.08	94.92	100
3σ	5.22	94.78	100	5.03	94.97	100
4σ	5.24	94.76	100	5.09	94.91	100

Supplementary Table 2: Proportion of observed intra- or interchromosomal transcript pairs for all 2.76M transcript pairs, and pairs above each standard deviation threshold.

	transcript_ID	Gene_ID	GO_ID	Term	Annotated	Significant	Expected	Fisher
1	ILMN_1772155	ZNF536	GO:0034048	negative regulation of protein phosphata...	2	2	0.04	3.4e-04
2	ILMN_2352303	RASSF2	GO:0006295	nucleotide-excision repair DNA incision...	2	2	0.04	3.1e-04
3	ILMN_1855355	HS.463489	GO:0021943	formation of radial glial scaffolds	3	2	0.05	8.1e-04
4	ILMN_2204215	HHIP	GO:0009650	UV protection	10	3	0.16	4.8e-04
5	ILMN_1708432	PLCB1	GO:0051289	protein homotetramerization	50	6	0.87	2.2e-04
6	ILMN_1745276	LOC730262	GO:0021987	cerebral cortex development	103	8	1.64	4.3e-04
7	ILMN_1791346	ATF3	GO:0034975	protein folding in endoplasmic reticulum	8	3	0.13	2.1e-04
8	ILMN_1779228	CDH2	GO:0060088	auditory receptor cell stereocilium orga...	11	3	0.19	7.3e-04
9	ILMN_1681598	PCDHGB7	GO:0022904	respiratory electron transport chain	100	8	1.66	3.1e-04
10	ILMN_2293744	MARVELD2	GO:0021747	cochlear nucleus development	4	3	0.06	1.6e-05
11	ILMN_1803652	C9ORF91	GO:0042100	B cell proliferation	80	6	1.33	8.1e-04
12	ILMN_2073409	LOC647215	GO:0043627	response to estrogen	200	8	3.10	3.6e-04
13	ILMN_1679139	HORMAD2	GO:0050776	regulation of immune response	882	21	14.05	1.2e-05
14	ILMN_1695282	RAPGEF1	GO:0043311	positive regulation of eosinophil degran...	2	2	0.03	2.4e-04
15	ILMN_1811367	MAT2B	GO:2001223	negative regulation of neuron migration	11	3	0.17	5.1e-04
16	ILMN_2396991	HCST	GO:0001975	response to amphetamine	28	5	0.40	4.0e-05
17	ILMN_1817438	HS.66072	GO:2001271	negative regulation of cysteine-type end...	3	2	0.05	7.2e-04
18	ILMN_1684886	VCX	GO:0034769	basement membrane disassembly	2	2	0.03	2.5e-04
19	ILMN_1759649	FLJ41352	GO:0006968	cellular defense response	48	5	0.71	7.1e-04
20	ILMN_2072622	FLJ12684	GO:0031573	intra-S DNA damage checkpoint	11	3	0.16	4.9e-04
21	ILMN_2131293	ALG1L	GO:0010976	positive regulation of neuron projection...	210	9	2.99	1.1e-04
22	ILMN_2387599	C20ORF24	GO:0045348	positive regulation of MHC class II bios...	8	3	0.11	1.3e-04
23	ILMN_1788160	KIF5B	GO:0006601	creatine biosynthetic process	2	2	0.03	2.3e-04
24	ILMN_1746856	RAB21	GO:0071872	cellular response to epinephrine stimulu...	12	3	0.18	6.2e-04
25	ILMN_2401701	PCGF6	GO:0086091	regulation of heart rate by cardiac cond...	26	4	0.37	4.7e-04
26	ILMN_1700307	FLJ38969	GO:0043508	negative regulation of JUN kinase activi...	12	3	0.17	5.9e-04
27	ILMN_1696119	SRI	GO:0060161	positive regulation of dopamine receptor...	3	2	0.04	6.1e-04
28	ILMN_1807298	NR4A3	GO:0035928	rRNA import into mitochondrion	2	2	0.03	1.9e-04
29	ILMN_1859207	HS.551143	GO:0071420	cellular response to histamine	8	3	0.12	1.7e-04
30	ILMN_1874362	HS.541829	GO:0042428	serotonin metabolic process	10	3	0.14	3.1e-04
31	ILMN_1668946	USH1C	GO:0019276	UDP-N-acetylgalactosamine metabolic proc...	3	2	0.04	6.4e-04
32	ILMN_1677153	OPN1LW	GO:0071872	cellular response to epinephrine stimulu...	12	3	0.18	6.5e-04
33	ILMN_2198393	KATNA1	GO:0048254	snoRNA localization	3	2	0.05	6.8e-04
34	ILMN_1767377	LOC153561	GO:0042110	T cell activation	400	13	5.84	2.0e-04
35	ILMN_1676891	CDC2L6	GO:0034048	negative regulation of protein phosphata...	2	2	0.03	2.0e-04
36	ILMN_1720771	STX11	GO:0006990	positive regulation of transcription fro...	4	2	0.06	1.3e-03
37	ILMN_1747223	FRYL	GO:0043311	positive regulation of eosinophil degran...	2	2	0.03	2.1e-04
38	ILMN_2150095	CES4	GO:0045348	positive regulation of MHC class II bios...	8	3	0.11	1.3e-04
39	ILMN_1813590	KIAA1026	GO:0031047	gene silencing by RNA	125	7	1.82	1.1e-03
40	ILMN_1741812	C6ORF78	GO:0006968	cellular defense response	48	6	0.68	5.7e-05
41	ILMN_2150586	ZNF536	GO:0034141	positive regulation of toll-like recepto...	7	3	0.10	9.0e-05
42	ILMN_1723007	ZCCHC9	GO:0071294	cellular response to zinc ion	16	3	0.23	1.5e-03
43	ILMN_1806533	PDE7B	GO:0031629	synaptic vesicle fusion to presynaptic a...	15	4	0.21	4.6e-05
44	ILMN_1804935	VNN3	GO:0086091	regulation of heart rate by cardiac cond...	26	5	0.37	2.9e-05
45	ILMN_2067453	LCAP	GO:0032230	positive regulation of synaptic transmis...	12	3	0.18	6.1e-04
46	ILMN_1770260	NFKBIZ	GO:0019276	UDP-N-acetylgalactosamine metabolic proc...	3	2	0.04	5.5e-04
47	ILMN_1807914	ACSL5	GO:0032230	positive regulation of synaptic transmis...	12	3	0.18	6.3e-04
48	ILMN_1712088	CLYBL	GO:0031573	intra-S DNA damage checkpoint	11	3	0.17	5.1e-04
49	ILMN_1686846	AKAP12	GO:0006915	apoptotic process	1685	36	23.67	4.7e-04
50	ILMN_1693552	CD300A	GO:0021943	formation of radial glial scaffolds	3	2	0.04	5.8e-04

Supplementary Table 3: Top GO terms for the 50 transcripts with the highest connectivity $\pm 3\sigma$.

AHDC1	HIST1H2BD	POLR1E	WHSC1L1
ANKRD32	HIST1H2BG	POLR2L	XBP1
ARID1A	HLA-DQB1	POLR3F	YEATS4
ARID4A	HMGN1	POU2AF1	ZBTB16
ARNTL	HMGN4	POU5F1P1	ZBTB20
ASB6	HOXC5	PRDM14	ZBTB25
ASCC2	HOXD13	PRDM2	ZBTB26
ATF3	ID3	RAD51	ZBTB32
ATF4	IKZF1	RERE	ZBTB44
ATF6	IKZF3	RFC1	ZDHHC13
ATMIN	IRF5	RNF14	ZDHHC7
BAZ1A	IRX3	RUNX2	ZFP90
BCL11A	JMJD2C	RUVBL1	ZIC5
BOLA2	KLF14	RXRG	ZMAT2
BRWD1	LARP1	RYBP	ZNF114
BTBD11	LASS5	SAFB	ZNF138
CAMTA2	LBXCOR1	SATB2	ZNF154
CBX3	LEF1	SCAND1	ZNF193
CD36	LHX5	SF1	ZNF22
CIITA	LMO3	SIRT1	ZNF230
CRAMP1L	LMX1A	SIRT5	ZNF239
CREB1	LYL1	SLA2	ZNF266
CREB3L2	MBNL3	SLC22A4	ZNF276
CRSP9	MCM6	SLC2A4RG	ZNF28
CSDA	MDS1	SMAD5	ZNF280D
CSDE1	MED4	SMARCA5	ZNF296
CUL5	MED6	SMARCD3	ZNF318
CUX1	MEF2D	SMARCE1	ZNF322B
DAZAP2	MGMT	SND1	ZNF333
DEK	MLL4	SOX10	ZNF343
DVL1	MSC	SOX11	ZNF365
E2F5	NCOA1	SPI1	ZNF391
EBF1	NCOA4	SPIB	ZNF415
EDF1	NEIL2	SPIC	ZNF423
ELF1	NFATC1	SREBF1	ZNF430
ENO1	NFKBIZ	SSH2	ZNF443
EPC1	NFXL1	STAT3	ZNF502
ERCC8	NFYA	STAT4	ZNF536
ETS2	NKX3-1	SUPT3H	ZNF555
EYA2	NOC4L	SUPT4H1	ZNF565
FAM171B	NR1H3	TAF1C	ZNF567
FBXO7	NR2C2	TAF5L	ZNF586
FLJ11795	NR4A3	TAF6	ZNF589
FOXK1	NRL	TCEA2	ZNF618
FOXL1	NUFIP1	TCEA3	ZNF624
FUBP3	ONECUT1	TCF12	ZNF626
FXR1	PAPOLA	TCF2	ZNF641
GATA4	PAX3	TCOF1	ZNF675
GATAD2A	PAX8	TFAP2A	ZNF679
GCM1	PCBD1	TFAP2B	ZNF681
GRHL3	PCGF6	THAP4	ZNF711
GTF2A2	PHF17	THAP9	ZNF792
H1F0	PHF6	THRAP3	ZNF80
H1FX	PHF7	**TSC22D1***	ZNF91
HCLS1	PHOX2B	**UBTF***	ZNF93
HES4	PLEKHA4	USF1	ZSCAN10
HIST1H1C	PMS1	USP39	ZZZ3
HIST1H2BC	POGK	VHL	

Supplementary Table 4: List of transcription factors enriched in $\hat{r}_G$ pairs $\geq$ 0.48 (2σ) and 0.72 (3σ). The entries marked in bold with asterisks are enriched at 3σ only.
